# Genotype–phenotype correlation in contactin-associated protein-like 2 (*CNTNAP-2*) developmental disorder

**DOI:** 10.1007/s00439-023-02552-2

**Published:** 2023-05-14

**Authors:** Gianluca D’Onofrio, Andrea Accogli, Mariasavina Severino, Haluk Caliskan, Tomislav Kokotović, Antonela Blazekovic, Kristina Gotovac Jercic, Silvana Markovic, Tamara Zigman, Krnjak Goran, Nina Barišić, Vlasta Duranovic, Ana Ban, Fran Borovecki, Danijela Petković Ramadža, Ivo Barić, Walid Fazeli, Peter Herkenrath, Carla Marini, Roberta Vittorini, Vykuntaraju Gowda, Arjan Bouman, Clarissa Rocca, Issam Azmi Alkhawaja, Bibi Nazia Murtaza, Malik Mujaddad Ur Rehman, Chadi Al Alam, Gisele Nader, Maria Margherita Mancardi, Thea Giacomini, Siddharth Srivastava, Javeria Raza Alvi, Hoda Tomoum, Sara Matricardi, Michele Iacomino, Antonella Riva, Marcello Scala, Francesca Madia, Angela Pistorio, Vincenzo Salpietro, Carlo Minetti, Jean-Baptiste Rivière, Myriam Srour, Stephanie Efthymiou, Reza Maroofian, Henry Houlden, Sonja Catherine Vernes, Federico Zara, Pasquale Striano, Vanja Nagy

**Affiliations:** 1 Department of Neurosciences Rehabilitation, Ophthalmology, Genetics, Maternal and Child Health (DiNOGMI), University of Genoa, Via Gerolamo Gaslini 5, 16147 Genoa, Italy; 2 Division of Medical Genetics, Department of Specialized Medicine, Montreal Children’s Hospital, McGill University Health Centre (MUHC), Montreal, Canada; 3 Department of Human Genetics, McGill University, Montreal, QC Canada; 4 Neuroradiology Unit, IRCCS Istituto Giannina Gaslini, Genoa, Italy; 5 Ludwig Boltzmann Institute for Rare and Undiagnosed Diseases, Vienna, Austria; 6 CeMM Research Center for Molecular Medicine of the Austrian Academy of Sciences, Vienna, Austria; 7 Department for Functional Genomics, Center for Translational and Clinical Research, University of Zagreb School of Medicine, Zagreb University Hospital Center, Zagreb, Croatia; 8 Department of Neurology, University Hospital Center Zagreb, University of Zagreb School of Medicine, 10000 Zagreb, Croatia; 9 Department of Pediatrics, General Hospital dr. Tomislav Bardek Koprivnica, Koprivnica, Croatia; 10 Department of Paediatrics, University Hospital Center Zagreb and University of Zagreb School of Medicine, Zagreb, Croatia; 11 Department of Pediatrics, Varazdin General Hospital, Varazdin, Croatia; 12 Division of Neuropediatrics, Department of Pediatrics, Children’s Hospital Zagre, Zagreb, Croatia; 13 Department of Pediatric Neurology, University Hospital Bonn, Bonn, Germany; 14 Child Neurology and Psychiatry Unit Children’s Hospital “G. Salesi” Azienda Ospedaliero-Universitaria delle Marche Ancona, Ancona, Italy; 15 Department of Pediatrics, Regina Margherita Children’s Hospital, Turin, Italy; 16 Department of Pediatric Neurology, Indira Gandhi Institute of Child Health, Bangalore, India; 17 Department of Clinical Genetics, Erasmus MC University Medical Center, Rotterdam, The Netherlands; 18 Department of Neuromuscular Disorders, UCL Institute of Neurology, Queen Square, London, WC1N 3BG UK; 19 Pediatric Neurology Unit, Pediatric Department, Albashir Hospital, Amman, Jordan; 20 Department of Zoology, Abbottabad University of Science and Technology, Abbottabad, Pakistan; 21 Department of Microbiology, Abbottabad University of Science and Technology KP, Abbottabad, Pakistan; 22 Division of Pediatric Neurology, Department of Pediatrics, American University of Beirut Medical Center, Beirut, Lebanon; 23 Unit of Child Neuropsychiatry, Department of Clinical and Surgical Neuroscience and Rehabilitation, Epilepsy Center, EPICARE Reference Network, IRCCS Giannina Gaslini, Genoa, Italy; 24 Department of Neurology, Johns Hopkins University School of Medicine, Baltimore, MD USA; 25 Department of Pediatric Neurology, Institute of Child Health, Children’s Hospital Lahore, Lahore, Pakistan; 26 Department of Pediatrics, Ain Shams University, Cairo, Egypt; 27 Department of Pediatrics, University of Chieti, Chieti, Italy; 28 Unit of Medical Genetics-IRCCS Istituto Giannina Gaslini, Genoa, Italy; 29 Epidemiology and Biostatistics Unit, IRCCS Istituto Giannina Gaslini, Genoa, Italy; 30 Department of Human Genetics, Faculty of Medicine, McGill University, Montreal, QC H3A 1B1 Canada; 31 Bioinformatics Platform, Research Institute of the McGill University Health Centre, Montréal, QC H4A 3J1 Canada; 32 Research Institute, McGill University Health Centre, Montreal, QC Canada; 33 Division of Pediatric Neurology, Department of Pediatrics, McGill University, Montreal, QC Canada; 34 School of Biology, The University of St Andrews, Fife, UK; 35 Neurogenetics of Vocal Communication, Max Planck Institute for Psycholinguistics, Nijmegen, The Netherlands; 36 Pediatric Neurology and Muscular Diseases Unit, IRCCS Istituto “Giannina Gaslini”, Via Gerolamo Gaslini 5, 16147 Genoa, Italy

## Abstract

Contactin-associated protein-like 2 (*CNTNAP2*) gene encodes for CASPR2, a presynaptic type 1 transmembrane protein, involved in cell–cell adhesion and synaptic interactions. Biallelic *CNTNAP2* loss has been associated with “Pitt-Hopkins-like syndrome-1” (MIM#610042), while the pathogenic role of heterozygous variants remains controversial. We report 22 novel patients harboring mono- (*n* = 2) and bi-allelic (*n* = 20) *CNTNAP2* variants and carried out a literature review to characterize the genotype–phenotype correlation. Patients (M:F 14:8) were aged between 3 and 19 years and affected by global developmental delay (GDD) (*n* = 21), moderate to profound intellectual disability (*n* = 17) and epilepsy (*n* = 21). Seizures mainly started in the first two years of life (median 22.5 months). Antiseizure medications were successful in controlling the seizures in about two-thirds of the patients. Autism spectrum disorder (ASD) and/or other neuropsychiatric comorbidities were present in nine patients (40.9%). Nonspecific midline brain anomalies were noted in most patients while focal signal abnormalities in the temporal lobes were noted in three subjects. Genotype–phenotype correlation was performed by also including 50 previously published patients (15 mono- and 35 bi-allelic variants). Overall, GDD (*p* < 0.0001), epilepsy (*p* < 0.0001), hyporeflexia (*p* = 0.012), ASD (*p* = 0.009), language impairment (*p* = 0.020) and severe cognitive impairment (*p* = 0.031) were significantly associated with the presence of biallelic versus monoallelic variants. We have defined the main features associated with biallelic *CNTNAP2* variants, as severe cognitive impairment, epilepsy and behavioral abnormalities. We propose CASPR2-deficiency neurodevelopmental disorder as an exclusively recessive disease while the contribution of heterozygous variants is less likely to follow an autosomal dominant inheritance pattern.

**Supplementary Information:**

The online version contains supplementary material available at 10.1007/s00439-023-02552-2.

## Introduction

Contactin-associated protein-like 2 (*CNTNAP2*) is one of the largest genes in the human genome located on chromosome 7q35-36.1 (Nakabayashi and Scherer 2001). It encodes for CASPR2, a member of the neurexin superfamily of cell adhesion proteins (Poliak et al. 1999). CASPR2 is a presynaptic type 1 transmembrane protein, with a large extracellular and smaller intracellular portion that participates in cell–cell adhesion and synaptic interactions. *CNTNAP2* is expressed throughout the developing and adult central nervous system (CNS) (Peñagarikano 2011). Mouse studies have uncovered a role for CASPR2 in neuronal migration and postmitotic neuronal development (Canali et al. 2018; Fernandes et al. 2019). Experimental studies on knock-out mice and in human cell lines support the hypothesis that CASPR2 is involved in neuronal migration, myelination, and neuronal transmission with a reduction in both inhibitory GABAergic neuronal numbers and excitatory neurotransmission (Peñagarikano 2011).

A homozygous 1-bp deletion (c.3709delG) of *CNTNAP2* was initially detected in an Old Order Amish kindred, whose nine affected children exhibited mild motor delay until the onset of intractable seizures during infancy, which were followed by deterioration in learning and language abilities, and social behavior (Strauss et al. 2006). Three subjects showed unilateral cortical dysplasia of the anterior temporal lobe, and neuronal migration defects from brain specimen biopsies. Altogether, this neurological disorder was named cortical dysplasia-focal epilepsy (CDFE) syndrome (Strauss et al. 2006). Subsequently, homozygous or compound heterozygous variants and/or intragenic deletions within *CNTNAP2* were associated with Pitt-Hopkins like syndrome 1 (PTHSL1, MIM#610042), with variable features that included intellectual disability (ID), early seizure onset, regression of language ability, and hyper-breathing patterns (Strauss et al. 2006; Zweier et al. 2009; Smogavec et al. 2016). Given the lack of typical Pitt-Hopkins craniofacial features and hyper-breathing patterns in most patients, it has recently been proposed that biallelic loss of *CNTNAP2* results in a disorder called “CASPR2-deficiency neurodevelopmental disorder (NDD)”, which includes severe ID, early infantile seizures, language regression, variable presence of autistic features, hyporeflexia and ataxia (Rodenas-Cuadrado et al. 2016).

A growing body of literature over the last two decades underscored a possible role of heterozygous chromosomal translocations and deletions, single nucleotide polymorphisms (SNPs), and rare heterozygous variants of *CNTNAP2.* These were found in a wide array of neuropsychiatric disorders, such as autism spectrum disorder (ASD), schizophrenia, obsessive–compulsive disorder, Gilles de la Tourette syndrome, attention deficit hyperactivity disorder (ADHD), dyslexia, specific language impairment and stuttering (Verkerk et al. 2003; Arking et al. 2008; Friedman et al. 2008; Mikhail et al. 2011; Newbury et al. 2011; Ji et al. 2013; Centanni et al. 2015). However, heterozygous *CNTNAP2* variations are also present in the healthy population including healthy parents of children with either mono- or biallelic variants. Thus, the evidence for the role of heterozygous variants in *CNTNAP2* in neuropsychiatric disorders has yet to be clarified (Toma et al. 2018). The identification and description of new patients with *CNTNAP2* variants may further define the criteria of the syndrome and better characterize its genotype–phenotype correlation (Rodenas-Cuadrado et al. 2016). We report 22 patients harboring mono- or biallelic variants in *CNTNAP2* and show genotype–phenotype correlations by including a further 50 previously reported patients.

## Material and methods

### Patient recruitment

We recruited 22 previously unreported patients from 17 unrelated families carrying mono or biallelic variants in *CNTNAP2*. Patients were followed up at 16 centers worldwide for developmental and epileptic encephalopathy (DEE) and/or neurodevelopmental disorders. Genetic analyses were performed either in a diagnostic or research setting. Subsequently, they were enrolled using the international platform GeneMatcher (Sobreira et al. 2015).

Firstly, the respective referring clinicians were asked to fill in a spreadsheet with all clinical and genetic information for each patient (Online Resource). Secondly, all available clinical and genetic data, electroencephalography (EEG) and neuroradiological images were reviewed by expert pediatric neurologists, neuroradiologist and geneticists. Written informed consent was obtained from parents or guardians.

### Genetic testing

Most *CNTNAP2* variants were detected by epilepsy Next Generation Sequencing (NGS) panel (*n* = 11) or autism/ID NGS panel (*n* = 1). Exome sequencing (ES) (singleton *n* = 3; trios *n* = 7) was performed in the respective collaborating centers using different analysis platforms according to the BWA/GATK’s based pipelines. Targeted Sanger sequencing using standard methods was also performed either for verification of identified variants or segregation analysis. Sequencing methods and additional genetic analyses performed per individual are summarized in Online Resource. All variants were classified according to the ACMG/AMP criteria (Richards et al. 2015). *CNTNAP2* variants are listed according to the transcript NM_014141.6 and copy number variants (CNV) refer to the hg19/GRCh37 assembly.

### Literature review

We performed a literature review on MEDLINE (accessed by PubMed, updated to December 2022) with the search term “*CNTNAP2*” and “*CASPR2*”, including articles with reported pathogenic or likely pathogenic variants or variants of uncertain significance (VUS) in *CNTNAP2* that were suspected to contribute to the phenotype of patients. Patients with copy number variation (CNV) that encompassed other genes that were likely to contribute to the phenotype and/or reports without available clinical information were excluded. We also excluded reports of subjects with limited clinical information.

### Statistical analysis

We used descriptive analysis to characterize our cohort and previously published *CNTNAP2* patients. Based on both datasets, we compared the phenotypes of patients harboring a heterozygosity variant versus (vs.) patients with biallelic variants using chi-squared test or a Fisher’s exact test.

## Results

### Patients

We enrolled 22 patients (14 males) aged between 3 and 19 years (Table 1). Consanguinity was reported in six families (6/17, 35.3%); a family history of neurological diseases and/or disabilities was present in 11/17 subjects (64.7%).

**Table 1:** Summary of clinical and neuroradiological features in our cohort of patients with *CNTNAP2* pathogenic variants

	Family 1		Family 2			Family 3		Family 4	Family 5	Family 6	Family 7
Individual	#1	#2	#3	#4	#5	#6	#7	#8	#9	#10	#11
Gender	M	M	F	F	F	F	M	M	F	M	M
Ethnicity	Croatian Roma	Croatian Roma	Croatian Roma	Croatian Roma	Croatian Roma	Croatian Roma	Croatian Roma	Croatian Roma	German	Italian	Italian
Neurological family history	+	+	+	+	+	+	+	+	–	–	+
Consanguinity	–	–	–	–	–	–	–	–	–	–	–
Genetic test	ES	Targeted PCR	Panel	Panel	Panel	Panel	Panel	Panel	Panel	Panel	Panel
cDNA variant	c.1361_1362del	c.1361_1362del	c.1361_1362del	c.1361_1362del	c.1361_1362del	c.1361_1362del	c.1361_1362del	c.1361_1362del	c.252G > A; c.3331C > T	del c.98-?_402+?; delc.98-?_1348+?	c.1777+2T > C
Protein variant	p.Asn454 ArgfsTer24	p.Asn454 ArgfsTer24	p.Asn454 ArgfsTer24	p.Asn454 ArgfsTer24	p.Asn454 ArgfsTer24	p.Asn454 ArgfsTer24	p.Asn454 ArgfsTer24	p.Asn454 ArgfsTer24	p.Trp84* p.Gln1111*	–	–
Inheritance	Hmz	Hmz	Hmz	Hmz	Hmz	Hmz	Hmz	Hmz	Ch	Ch	Hmz
Dysmorphisms	–	–	+	–	–	+	+	–	–	–	+
Psychomotor delay	–	+	–	+	+	+	+	+	+	+	+
Developmental regression	+	–	+	–	–	–	–	–	–	–	–
ID	Profound	Severe	Severe	Moderate	Moderate	Moderate	Severe	Moderate	Mild	Mild	Severe
ASD	–	–	–	–	–	–	–	–	–	+	+
Other NPsy	–	–	Coprophagia, aggressivity	–	Coprophagia	Aggressivity	–	Agitation, hyperactivity	–	hyperactivity	-
Language impairment	+	+	+	+	+	+	+	+	+	+	+
Hypotonia	+	+	–	–	+	+	–	+	–	–	+
Hyporeflexia	+	+	–	–	+	–	–	+	–	–	–
Ataxia	+	–	–	–	+	+	–	–	–	+	–
Epilepsy	+	+	+	+	+	+	+	+	+	+	+
Neuroimaging:											
Normal	+	–	–	+	–	–	+	–	+	+	-
WM volume reduction	–	–	+	–	–	–	–	–	–	–	–
CC abnormalities	–	+	+	–	–	–	–	+	–	–	+
Cerebral atrophy	–	–	+	–	–	–	–	–	–	–	+
IVH	–	+	+	–	+	+	–	+	–	–	–
SCV atrophy	–	–	+	–	–	–	–	+	–	–	–
Temporal FCD	–	+	–	–	–	–	–	+	–	–	–
DN signal alterations	–	–	–	–	–	+	–	–	–	–	–
Comorbidity and/or complications	Aspiration pneumonia, feeding difficulties with failure to thrive	Rectum prolapse, hemolytic anaemia	Precocious puberty	Rectum prolapse, failure to thrive	–	–	Respiratory infections, pancytopenia, failure to thrive	–	–	–	Breathing disturbances, failure to thrive
Deceased (cause of death)	yes, 13 y (cachexia)	–	–	–	–	–	–	–	–	–	–

	Family 8	Family 9	Family 10	Family 11	Family 12	Family 13	Family 14		Family 15	Family 16	Family 17
Individual	#12	#13	#14	#15	#16	#17	#18	#19	#20	#21	#22
Gender	F	M	F	M	M	F	M	M	M	M	M
Ethnicity	Indian	Dutch	Arab-Jordanian	Punjabi	Lebanese	Italian	Egyptian	Egyptian	Egyptian	Italian	American white
Neurological family history	–	+	+	+	+	–	+	+	–	–	+
Consanguinity	+	–	+	+	+	–	+	+	+	–	–
Genetic test	WES	WES	WES	WES	WES	arrayCGH	WES	WES	WES	WES	Panel
cDNA variant	c.3407_3411del	c.400T > G; c.2449G > A	c.2151C > A	c.550+5G > T	c.1680del	del 7q35; del7q36.1	c.3262C > T	c.3262C > T	c.774_775insGGGCTGCC	c.3814A > T	c.1628delG
Protein variant	p.Tyr1136 SerfsTer27	p.Trp134Gly; p.Gly817Arg	p.Tyr717Ter	–	p.Asn561 IlefsTer45	-	p.Arg1088Ter	p.Arg1088Ter	p.Ter261ProfsTer?	p.Ile1272Phe	p-S5431fsX13
Inheritance	Hmz	Ch	Hmz	Hmz	Hmz	Ch	Hmz	Hmz	Hmz	Htz	Htz
Dysmorphisms	+	+	+	+	–	–	–	–	–	–	–
Psychomotor delay	+	+	+	+	+	+	+	+	+	+	–
Developmental regression	–	–	–	–	–	+	–	–	–	–	–
ID	Severe	Moderate	Severe	Moderate	Mild	Severe	Moderate	Moderate	Moderate	Mild	Unclear
ASD	+	+	+	+	–	+	–	–	–	+	+
Other NPsy	–	ADHD	–	–	–	Sleep disorder	–	–	–	Aggressivity, RLS	ADHD
Language impairment	+	+	+	+	+	+	+	+	+	+	+
Hypotonia	+	–	–	+ /–	+	+	–	–	±	–	+
Hyporeflexia	–	–	–	–	–	+	–	–	–	–	–
Ataxia	+	–	–	–	–	+	–	–	–	–	–
Epilepsy	+	+	+	+	+	+	+	+	+	+	–
Neuroimaging:											
Normal	–	–	–	+	–	–	+	+	+	+	Not performed
WM volume reduction	–	+	–	–	+	–	–	–	–	–	
CC abnormalities	–	+	–	–	+	–	–	–	–	–	
Cerebral atrophy	–	–	–	–	–	–	–	–	–	–	
IVH	+	+	+	–	+	+	–	–	–	–	
SCV atrophy	+	–	–	–	–	+	–	–	–	–	
Temporal FCD	+	–	–	–	–	–	–	–	–	–	
DN signal alterations	+	–	–	–	–	–	–	–	–	–	
Comorbidity and/or complications	–	–	–	Increase risk of fractures, failure to thrive	–	Breathing disturbances, RGE	–	–	–	–	Asthma, HG, periorbital angioedema
Deceased (cause of death)	–	–	–	–	–	–	–	–	Yes, 7 y (unknown cause of death)	–	–

*aCGH array* comparative genomic hybridization, *ADHD* attention deficit hyperactivity disorder, *ASD* autism spectrum disorder, *CC* corpus callosum, *Ch* compound heterozygous, *DN* dentate nuclei, *ES* exome sequencing, *FCD* focal cortical dysplasia, *HG* hypogammaglobulinemia, *Hmz* homozygous, *Htz* heterozygosity, *ID* intellectual disability, *IVH* inferior vermis hypoplasia, *m* months, *MRI* magnetic resonance imaging, *NPsy* neuropsychiatric disorder, *RGE* gastroesophageal reflux, *RLS* restless legs syndrome, *y* years, *SCV* superior cerebellar vermis, *WM* white matter, # individual, + present, – not present

### Auxology and dysmorphology

**Fig. 1:**
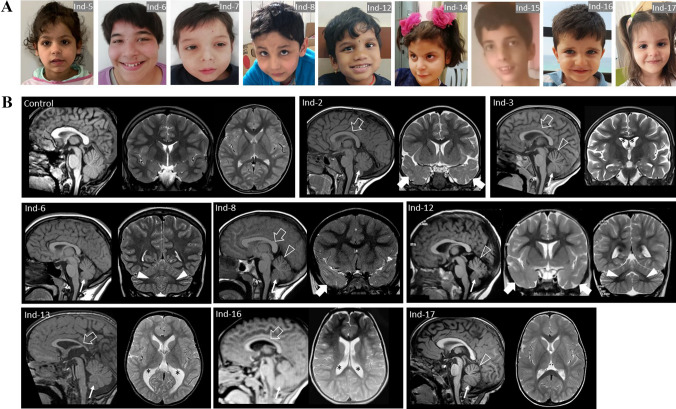
**a** Ind- 5, -6, -7, -8, -12, -14, -15, -16 and -17 iconography is shown (from left to right). Common facial dysmorphisms are shown including prominent ears (Ind-6 and Ind-7) and hypertelorism (Ind-6, Ind-12). Ind-7 shows mild ptosis of the left eyelid. Ind-14 presents with lips thickness, prognathism, and prominent philtrum. A lean, elongated face with mild lax skin is observed in Ind-15. Ind-17 has sparse hair. No noticeable dysmorphisms are appreciable in Ind-5, Ind-8, Ind-16 and Ind-17. **b** Brain MRI findings of the patients and a control; sagittal T1-weighted (first) and coronal and/or axial T2-weighted (middle and/or last) images. Inferior cerebellar vermis hypoplasia is noted in all the cases included in the figure (thin arrows) associated with mild superior cerebellar vermis atrophy in Ind-3, Ind-8, Ind-12, and Ind-17 (empty arrowheads). A thin corpus callosum is present in Ind-2, Ind-13 and Ind-16, while a thick posterior corpus callosum is noted in Ind-3 and Ind-8 (empty arrows). Mild white matter volume reduction with consequent ventricular enlargement is noted in Ind-3, Ind-13 and Ind-16 (asterisks). Cerebellar dentate nuclei T2 hyperintensity is visible in Ind-6 and Ind-12 (arrowheads). In Ind-2, Ind-8 and Ind-12 there are additional uni- or bilateral T2 hyperintensities at the level of the anterior temporal lobes (thick arrows) in keeping with focal cortical dysplasias

Five patients (5/22, 22.7%) presented with failure to thrive. Four subjects had microcephaly while one was found to be macrocephalic. A total of eight patients (8/22, 36.3%) exhibited non-specific facial dysmorphisms (Fig. 1a). In addition, *café-au-lait* stains were observed in two patients.

### Neurodevelopment

Global developmental delay (GDD), of variable severity, is reported in almost all patients (21/22, 95.5%). Moreover, individual-1 (Ind-1) and Ind-3 had early normal development before the onset of epilepsy, leading to major irreversible regression, while Ind-17 experienced a partial recovery of her cognitive and motor skills after seizure control. Intellectual disability (ID) has been assessed as mild in 4 patients, moderate in 9, severe in 7 and profound in 1 subject, whereas Ind-22 had a borderline intelligence quotient.

### Epilepsy

**Fig. 2:**
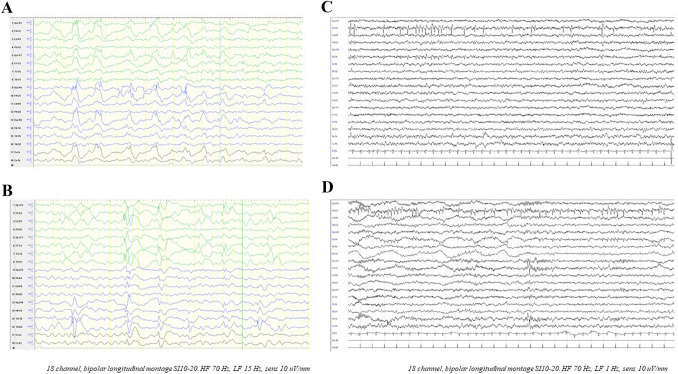
EEG features. **a**. Ind-1, 10 years old. Sleep recording. High voltage bilateral anterior delta waves and focal spikes over the frontal regions of both hemispheres. **b** Ind-2, 2 years 9 months. Awake recording. Synchronous and asynchronous spikes on bilateral frontal–temporal regions. **c** Ind-10, 3 years old. Awake recording. Right central-temporal medium voltage sharp waves. **d** Ind-10, 3 years old. Sleep recording. Nearly sub-continuous trend of right central-temporal sharp waves in the N2 phase, with a tendency to spread

Epilepsy occurred in 21 patients (95.5%) with onset at median age of 22.5 months [17 25th percentile–29.2 75th percentile]. Major findings are summarized in Table 2. Seizures were mainly described as primary generalized tonic–clonic (GTC) seizures (11/21, 52.3%) or focal motor seizures with impaired awareness (FIA) (11/21, 52.3%) and focal to bilateral (7/21, 33.3%). Tonic seizures (5/21, 23.8%), absences (3/21, 14.3%) and atonic seizures (2/21, 9.5%) were also reported. In three patients fever represented a trigger (3/21, 14.3%). Status epilepticus has occurred in 2 individuals (2/21, 9.5%). Half of the cohort experienced daily seizures at onset (10/21, 47.6%). Median number of anti-seizure medications (ASMs), prescribed over the course of their history, was 3 [3 25th percentile–5 75th percentile]. Eight patients (8/21, 38%) achieved seizure freedom for more than one year, and the other 8 (8/21, 38%) benefited from ASMs by showing a considerable seizure frequency reduction greater than 50%. None of them discontinued ASMs nor did any of them undergo epilepsy surgery. EEG often showed epileptic discharges in the temporal or fronto-temporal regions (8/21, 38%) (Fig. 2).

**Table 2:** Epilepsy overview in our cohort of patients with *CNTNAP2* pathogenic variants

	Family 1		Family 2			Family 3		Family 4	Family 5	Family 6
Individual	#1	#2	#3	#4	#5	#6	#7	#8	#9	#10
cDNA variant	c.1361_1362del	c.1361_1362del	c.1361_1362del	c.1361_1362del	c.1361_1362del	c.1361_1362del	c.1361_1362del	c.1361_1362del	c.252G > A; c.3331C > T	del c.98-?_402+?; delc.98-?_1348+?
Protein variant	p.Asn454 ArgfsTer24	p.Asn454 ArgfsTer24	p.Asn454 ArgfsTer24	p.Asn454 ArgfsTer24	p.Asn454 ArgfsTer24	p.Asn454 ArgfsTer24	p.Asn454 ArgfsTer24	p.Asn454 ArgfsTer24	p.Trp84* p.Gln1111*	–
Inheritance	Hmz	Hmz	Hmz	Hmz	Hmz	Hmz	Hmz	Hmz	Ch	Ch
Epilepsy onset	2 y	21 m	15 m	20 m	9 m	2 y	2 y	2 y 11 m	21 m	2 aa 9 m
Febrile seizures	–	–	–	–	–	–	–	–	–	+
Seizure types	FIA, GTC	GTC, FIA	GTC	FIA	FA, FIA, FBTC, SE	FBTC, GTC	GTC, Ton, SE	FA, FIA, FBTC	GTC, FBTC	FIA, FBTC
Seizure frequency	> 1/day	> 1/day	> 1/day	> 1/day	> 1/week	> 1/day	> 1/day	Occasionally	> 1/week	> 1/week
EEG	In sleep, high voltage anterior delta waves and focal spikes F-T regions	Frontal delta waves, focal spikes over left, and less prominent over right F-T regions	S-W in left F-T lobe with tendency to diffuse (15 m); theta-delta activity without epileptiform abnormalities (5 y)	Theta-delta activity, left posterior ictal epileptic discharges with diffusion	Slow focal epileptic discharges over both temporal lobes	Focal epileptic discharges in central regions	Background activity with irregular voltages	Initially normal, then right temporal epileptic discharges; normalization with ASM	Initially normal, then multifocal epileptic discharges both hemispheres independently	Right centro-temporal S-W complexes
ASMs and/or other non-pharmacological treatments	VPA, CBZ, PB, TPM, LTG, CNZ	PB, CBZ, VPA, TPM, LEV	VPA, LEV, CLB	VPA, LEV, CNZ, PHT	VPA, LEV, OXC	VPA, TPM, CBZ, LTG, PHT, OXC, TPM, LEV	VPA, LTG	VPA, OXC, LCS, ZNS	LEV, VPA, LCS, CLB	PHT, VPA, CNZ, CBZ, CLB, LCS, LTG
Response to treatment	Seizure free 2 y with TPM	Seizure free 4 m with CBZ	Good control	Partial control	Good	Seizure free with CBZ and LEV	Good	More than > 1 y seizure free	Seizure free with VPA and LCS	Good with LTG add-on to VPA

	Family 7	Family 8	Family 9	Family 10	Family 11	Family 12	Family 13	Family 14		Family 15	Family 16
Individual	#11	#12	#13	#14	#15	#16	#17	#18	#19	#20	#21
cDNA variant	c.1777+2T > C	.3407_3411del	c.400 T > G; c.2449G > A	c.2151C > A	c.550 + 5G > T	c.1680del	del 7q35; del7q36.1	c.3262C > T	c.3262C > T	c.774_775ins GGGCTGCC	c.3814A > T
Protein variant	–	p.Tyr1136 SerfsTer27	p.Trp134Gly; p.Gly817Arg	p.Tyr717Ter	–	p.Asn561IlefsTer45	–	p.Arg1088Ter	p.Arg1088Ter	p.Ter261ProfsTer?	p.Ile1272 Phe
Inheritance	Hmz	Hmz	Ch	Hmz	Hmz	Hmz	Ch	Hmz	Hmz	Hmz	Htz
Epilepsy onset	17 m	2 y	4.5 y	31 m	2.5 y	17 m	17 m	27 m	13 m	31 m	1 m
Febrile seizures	–	+	–	–	–	–	+	–	–	–	–
Seizure types	Ton, FIA, GTC, A	GTC, FIA	GTC, Ab	Ton, A	FIA	FIA, Ab	FA, FIA, FBTC	GTC	FBTC	Ton, GTC	Ton, Ab
Seizure frequency	> 1/week	> 1/week	Occasionally	> 1/day	> 1/day	> 1/week	> 1/day	> 1/day	> 1/month	> 1/month	Occasionally
EEG	Slow (< 2.5 Hz) S-W e and paroxysmal fast activity in slow sleep (Lennox-like)	Left focal epileptic discharges	Multifocal epileptic discharges	No epileptiform activity	Generalized high amplitude burst sharp and slow waves	Left central and frontal spikes	Alternating side ictal pattern involving the F-T regions	Normal	Normal	Normal	Bilateral synchronous slow sharp discharges over the posterior areas
ASMs and/or other non-pharmacological treatments	VPA, CBZ	VPA, CLB, LEV	VPA, LTG	VPA, CBZ, CNZ, LEV, TPM	VPA, LTG, CBZ	OXC, VPA	PB, VPA, CBZ, CLB, LEV, CNZ, TPM, LCS, KD, ZNS	LEV, OXC, VPA	CBZ, VPA, TPM, LEV, OXC, PHT	VPA, LEV, TPM	VPA
Response to treatment	Partial	Partial	Seizure free	Partial efficacy of LEV and VPA, optimal control with TPM; seizure free from 5 y	Reduced frequency to > 1/month; seizure free for maximum 6 m	Good control on OXC	Better seizure control and progressive achievement of seizure freedom with ZNS	Seizure free since age of 3 y	Partial (decrease in number and duration)	No control	Good to VPA

*A* atonic, *Ab* absences, *ASMs* anti-seizure medications, *BRV* brivaracetam, *CBZ* carbamazepine, *Ch* compound heterozygous, *CLB* clobazam, *CNZ* clonazepam, *EEG* electroencephalography, *F* frontal, *FA* focal aware, *FBTC* focal to bilateral tonic–clonic, *FIA* focal impaired awareness, *F-T* fronto-temporal, *GTC* generalized tonic–clonic, *Hmz* homozygous, *Htz* heterozygosity, *m* months, *KD* ketogenic diet, *LCS* lacosamide, *LEV* levetiracetam, *LTG* lamotrigine, *MED* multifocal epileptiform discharges, *OXC* oxcarbazepine, *P* parietal, *PB* phenobarbital, *PER* perampanel, *PHT* phenytoin, *RFN* rufinamide, *SE* status epilepticus, *S-W* spike wave, *T* temporal, *Ton* tonic, *TPM* topiramate, *VPA* valproate, *ZNS* zonisamide, *y* years, # individual, > more than, + present, *NA* not available, – not present

### Neuropsychiatric features and other neurological and neurobehavioral findings

Expressive and/or receptive language was consistenly impaired in all patients. A formal diagnosis of ASD was reported in nine patients (9/22, 40.9%), variably associated with other neuropsychiatric comorbidities such as hyperactivity (4/21, 19%) and behavioral issues (4/21, 19%). More specifically, sudden episodes of aggressive and violent behavior were reported in Ind-3, Ind-6 and Ind-21, while psychomotor agitation occurred occasionally in Ind-8. Coprophagia was reported in two sisters (2/22, 9.1%) from family 2. No other psychiatric comorbidities have been identified in our population. Neurological examination revealed hypotonia of varying degrees in 12 cases (12/22, 55%) and hyporeflexia in 5 (5/22, 23%). Six patients exhibited an ataxic gait (6/22, 27%). Two patients presented with breathing disorders consisting of episodes of hyperpnea and apnea during the day (2/22, 9%). No sensorineural deficits or extrapyramidal disorders were noted.

### Neuroimaging

Neuroimaging studies were performed in 21/22 subjects, including 18 brain magnetic resonance imaging (MRI) and 3 computed tomography (CT) studies (Ind-1, Ind-5 and Ind-7). Brain MRI revealed non-specific dysmorphisms in the majority of subjects (11/21, 52.4%) (Fig. 1b), including inferior cerebellar vermis hypoplasia (9/21, 42.9%), abnormalities of the corpus callosum (6/21, 28.5%; thick in two cases and thin in four other cases), superior cerebellar vermis atrophy (4/21, 19%), mild white matter volume reduction with ventricular enlargement (3/21, 14.3%), cerebellar dentate nuclei signal alterations (2/21, 9.5%), and mild cerebral atrophy (2/21, 9.5%). Signal abnormalities consistent with focal cortical dysplasia were noted at the level of the anterior temporal lobes in three subjects (Ind-2, Ind-8, and Ind-12). Neuroimaging was unremarkable in ten patients (10/21, 47.6%).

### Other comorbidities

Extra-neurological comorbidities occurred in nine individuals (9/22, 40.9%), including recurrent respiratory infections, haematological disorders (pancytopenia, haemolytic anaemia) and rectal prolapse (2/22, 9%). Precocious puberty, asthma, hypogammaglobulinemia, gastroesophageal reflux and osteopenia were reported once (1/22, 4.5%). None of our patients presented with congenital abnormalities of any extra-CNS organ. Two patients in our cohort deceased: the first at the age of 13 (Ind-1) due to cachexia in the context of feeding difficulties and severe GDD, while the other (Ind-20) at 7 years for unknown reasons. No statistically significant differences were observed when comparing patients with a history of consanguinity and non-consanguinity.

### Genetic results

A total of 18 distinct *CNTNAP2* variants were identified, seven of which were novel (Online Resource). Except for two heterozygous variants, all other individuals were found to harbor biallelic variants; either homozygous (*n* = 16) or compound heterozygous (*n* = 4) variants. Variants included ten likely gene-disrupting (LGD) variants, four intragenic deletions (identified either by microarray or an epilepsy NSG gene panel) and three missense variants. Sanger sequencing confirmed variants segregation with the phenotype within these families. All variants were absent or extremely rare in human population variant databases (allele frequency ranging from 0 to 0.0001557 in the gnomAD database). None of the variants were reported in a homozygous state in healthy individuals. LGD variants were scattered throughout *CNTNAP2* and included four different frameshift and four nonsense changes and two splice site variants. All frameshift and nonsense variants were predicted to result in premature termination codon and, therefore, likely be degraded through nonsense-mediated mRNA decay (NMD). As such these were classified as pathogenic/likely pathogenic. Of note, the frameshift variant c.1361_1362del p.(Asn454ArgfsTer24) was recurrent in eight subjects of families 1–4 of Croatian Roman ancestry and the nonsense variant c.3262C > Tp.(Arg1088Ter) was found in three subjects of two nonrelated Egyptian families suggesting these variants are likely to be founder mutations in these populations. Two individuals carried homozygous splicing variants as follows: the variant c.1777+2T > C (Ind-11) affects the consensus GT-splice donor site of intron 11 and was computationally predicted to cause a loss of a splice donor site disrupting the reading frame and resulting in NMD (Splice AI score 0.98). Thus, it was classified as likely pathogenic according to the ACMG criteria. The homozygous variant c.550+5G > T (Ind-15) predicts a loss of a splice donor site (Splice AI score 0.66), yet it remains a VUS according to the current ACMG guidelines. CGH-array revealed two intragenic *CNTNAP2* deletions in Ind-17: a 31,949 bp deletion in 7q35(147,651,818–147,683,766) encompassing exon 15 and inherited by her mother and a paternally inherited deletion of 9317 bp in 7q36.1 (148,071,316–148,080,632), encompassing exon 22. These deletions were confirmed by multiplex ligation-dependent probe amplification. Epilepsy gene panel showed two compound heterozygous deletions in Ind-10, namely the c.98-?_402+? that encompasses exons 2–3 of *CNTNAP2* and the heterozygous deletion c.98-?_1348+?, encompassing exons 2–8. Both deletions were confirmed by CGH-array. Individual-13 harbored the compound heterozygous missense variant c.400T > G p.(Trp134Gly) and c.2449G > A p.(Gly817Arg) that were classified as VUS. We included subjects harboring these biallelic VUS given supporting criteria of pathogenicity and consistent phenotype. The heterozygous missense variant c.3814A > T p.(Ile1272Phe) was found to be de novo in individual 21 while an ID/ASD panel identified in Ind-22 the frameshift variant c.1628del p.(Ser543Ilefs*13) that was maternally inherited. Both variants were classified as VUS.

Overall, we ascertained a diagnosis with biallelic *CNTNAP2* pathogenic/likely pathogenic variants in 18 out of 22 subjects included in this study. No other pathogenic/likely pathogenic variants were identified in the currently known NDD-related genes in the ES data in these families. Additional VUS detected in our cohort either by ES or microarray are listed in Online Resource.

### Previously published cases

**Fig. 3:**
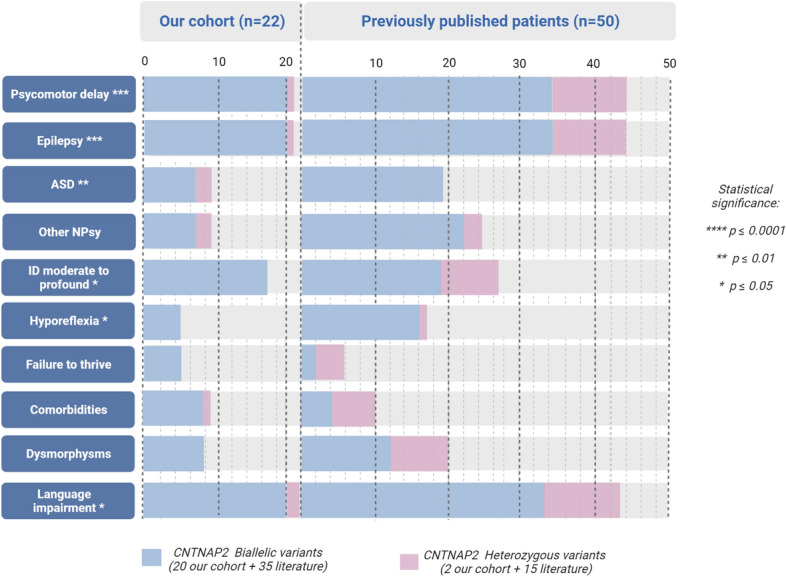
Summary of the key clinical features of patients carrying mono- or bi-allelic pathogenic *CNTNAP2* variants in our cohort and the literature. ID intellectual disability, NPsy neuropsychiatric findings. Statistical significance refers to patients with biallelic versus monoallelic variants

**Fig. 4:**
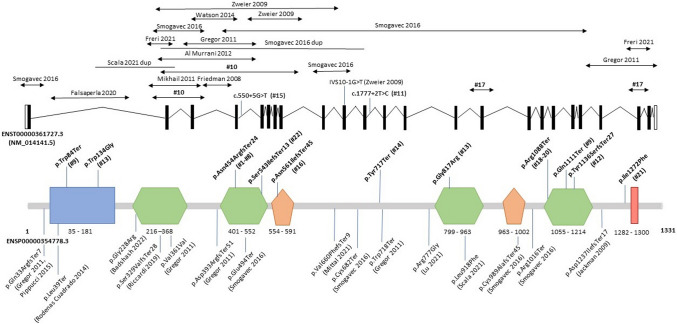
*CNTNAP2* variants position in our cohort (in bold, # individual) and previously published patients. The arrow indicates a deletion, and the line a duplication

We identified 50 previously published patients from 17 articles (Strauss et al. 2006; Friedman et al. 2008; Jackman et al. 2009; Zweier et al. 2009; Gregor et al. 2011; Al Murrani et al. 2012; Watson et al. 2014; Pippucci et al. 2015; Smogavec et al. 2016; Rodenas-Cuadrado et al. 2016; Riccardi et al. 2019; Falsaperla et al. 2020; Freri et al. 2021; Lu et al. 2021; Mittal et al. 2021; Scala et al. 2021; Badshash et al. 2022) reporting the clinical phenotype of patients carrying pathogenic or likely pathogenic *CNTNAP2* variants or VUS suspected to contribute to the phenotype (Online Resource). Figure 3 summarizes the main phenotypic features observed in our cohort and in the literature, distinguishing between heterozygous and homozygous variants, while variant positions are shown in Fig. 4.

### Genotype–phenotype correlation

Altogether, GDD and epilepsy were significantly more present in patients harboring homozygous variants than in heterozygous patients (*p* < 0.0001) (Online Resource). Similarly, ASD (*p* = 0.009), hyporeflexia (*p* = 0.012), language impairment (*p* = 0.020), as well as a moderate to severe degree of ID (*p* = 0.031) were more frequent in patients with biallelic variants.

## Discussion

We reported a cohort of 22 new patients harboring either biallelic (20) or monoallelic variants (2) in *CNTNAP2*. To the best of our knowledge, this is the largest cohort of patients with *CNTNAP2* variants reported together to date. Our study corroborates previous literature, confirming that *CNTNAP2* deficiency due to biallelic variants leads to a distinct neurodevelopmental disorder typically characterized by developmental delay, seizure onset within the first 2 years followed by developmental regression, moderate to severe ID and variable occurrence of ASD and behavioral abnormalities. Similarly to previous reports, hypotonia and hyporeflexia are frequent, whereas only a few patients display ataxia. Likewise, occipital frontal circumference is normal in the majority of patients in contrast to the initial reports of relative macrocephaly. Furthermore, our patients harboring biallelic variants do not display the typical craniofacial features and abnormal breathing patterns reported for PTHS. Together, this supports previous literature suggesting that the name PTHS1 should be replaced by CASPR2-deficiency NDD (Rodenas-Cuadrado et al. 2016). In addition, the occurrence of epilepsy in virtually all patients within the first 2 years with consequent regression of development and cognitive impairment would suggest a DEE. Epilepsy is indeed a cardinal feature in patients with biallelic *CNTNAP2* variants. The onset of seizures typically occurs in the first two to three years of life. Seizures initially are very frequent and difficult to treat. However, most patients achieve good seizure control within a few years after onset. Seizures are most frequently focal motor, at times with secondary generalization This is in line with previous descriptions in the literature (Strauss et al. 2006; Rodenas-Cuadrado et al. 2016; Smogavec et al. 2016). Cortical areas most typically involved seem to be the frontal and temporal regions (Strauss et al. 2006).

CASPR2 is found in the inhibitory presynaptic compartment and, to a lesser extent, in the excitatory postsynaptic compartment where it is involved in several pivotal processes, such as neurite development and synapse maturation, stability, and function (Horresh et al. 2008). It also localizes to juxtaparanodes of myelinated axons, where it is involved in neuron-glia interactions, and mediates the clustering of potassium channels via interaction with contactin 2 (also known as TAG-1) (Horresh et al. 2008). Similar to humans, *Cntnap2*−/−mice display epilepsy in addition to ASD features and cortical developmental abnormalities (Peñagarikano et al. 2011). RNAi-mediated knock-down of Caspr2 produced a cell-autonomous decrease in dendritic arborization and spine development in pyramidal neurons, decreasing the number of excitatory and inhibitory synapse numbers, and impairing synaptic transmission (Anderson et al. 2012). Together, these observations suggest that a perturbation of synaptic homeostasis and function due to CASPR2 deficiency leads to an imbalance of excitatory and inhibitory post-synaptic currents in neural networks that may contribute to epilepsy phenotypes (Anderson et al. 2012).

Strauss et al. (2006) described neuroimaging features of focal cortical dysplasia in three subjects that were consistent with findings of neuronal migration defects from brain biopsies. These results were in line with neuropathological and physiological studies in the *Cntnap2*−/−mice showing neuronal migration abnormalities, reduced number of interneurons and abnormal neuronal network activity (Peñagarikano et al. 2011). Subsequent to this, no further reports have described malformations of cortical development: however, cerebellar hypoplasia and nonspecific white matter abnormalities have been occasionally reported in subjects with biallelic *CNTNAP2* variants (Zweier et al. 2009; Smogavec et al. 2016). Here, we describe the largest cohort of subjects for whom brain MRI was available, showing that three subjects had unilateral or bilateral anterior temporal lobe T2 hyperintensities consistent with focal cortical dysplasia, supporting the notion of malformation of cortical development due to *CNTNAP2*-deficiency. Interestingly, we also noted several nonspecific findings that have been described in subjects with PTHS, including callosal anomalies, white matter volume reduction, dentate nuclei signal alterations and other minor posterior fossa abnormalities.

All our patients suffered from severe speech impairment and one-third had ASD or other behavioral abnormalities including aggressive behavior and stereotypic movements. There is evidence that supports a role for *CNTNAP2* in language development, including enriched expression during human brain development in frontotemporal-subcortical circuits known to be critical for human executive function (Alarcón et al. 2008). Despite some conflicting results (Sampath et al. 2013; Murdoch et al. 2015; Toma et al. 2018; Zhang et al. 2019), several studies have linked SNPs in *CNTNAP2* variants with ASD and/or language-related disorders (Vernes et al. 2008; Li et al. 2010; Gregor et al. 2011; Uddin et al. 2021). Further, some SNPs (e.g. rs2710102 and rs7794745) have been associated with abnormal activation of the right inferior frontal gyrus (Broca’s area homologue) and right lateral temporal cortex in subject with ASD and reduced volume of specific grey matter areas (Whalley et al. 2011). Together this evidence supports an impact of *CNTNAP2* variation on language related brain regions and phenotypes; however, it is not yet clear what role (if any) CASPR2 has in the development of language.

While the loss of function (LoF) mechanism due to biallelic *CNTNAP2* variants is well understood, the impact of heterozygous *CNTNAP2* variants is more controversial. It has been suggested that the phenotypic picture of each heterozygous variant may result from the combination of two mechanisms. On the one hand a dominant-negative effect on wild-type Caspr2 function might be due to endoplasmic reticulum (ER) retention mimicking the homozygous null phenotype (Canali and Goutebroze 2018). On the other hand a loss of function mechanism for adhesion-defective variant proteins, could enable the interaction with their extracellular partners (Canali and Goutebroze 2018). According to this model, the phenotype of our patient, Ind-21 (mild ID, epilepsy and behavioral abnormalities) harboring the de novo missense variant p.(Ile1272Phe) lying in the extracellular domains may be due to a LoF mechanism if the protein is secreted from the ER. However, the impact of the de novo frameshift variant in the patient (Ind-22) with isolated ASD remains controversial since it is predicted to undergo nonsense-mediated decay and thus it would unlikely exert a dominant negative effect. It is also noteworthy that *CNTNAP2* is not constrained for missense and Lof variants in the gnomAD databse (Z score − 0.29, pLI score 0) indicating that heterozygous missense and Lof variants of *CNTNAP2* are not subject to negative selection (Lek et al. 2016). This is in line with the fact that carrier parents of *CNTNAP2* variants are healthy. Furthermore, large scale studies on gene enriched for de novo variants in NDD have failed to highlight this gene with any meaningful significance (Kaplanis et al. 2020; Satterstrom et al. 2020) and several other studies did not identify a significant burden for CNTNAP2 rare variants in patients with ASD or schizophrenia comparing to controls (Murdoch et al. 2015; Toma et al. 2018; Zhang et al. 2019), suggesting that *CNTNAP2* is not a a primary risk gene for psychiatric disorders. Although it might be possible that *CNTNAP2* heterozygous variants contribute to ASD and related neuropsychiatric phenotypes with a polygenic inheritance pattern, it seems unlikely based on the above observations that they solely result in a neuropsychiatric phenotype following a classical autosomal dominant Mendelian inheritance. Taken together, we propose CNTNAP2-related NDD as an exclusively recessive disorder while the dominant version is becoming weaker with the increase body of evidence in the literature and in human population variant databases.

In conclusion, we report the largest cohort of patients with *CNTNAP2* variants to date and define the core phenotype associated with biallelic *CNTNAP2* variants. These data suggest that patients with biallelic variants are likely to develop severe cognitive impairment, epilepsy and variable behavioral abnormalities.

In most cases, patients have an unremarkable perinatal history and a normal psychomotor development or slightly delayed during the first year of life. Concomitant with the epilepsy onset, occurring more often during the second year of life, developmental stagnation or regression is observed. Epilepsy can be difficult to control at the beginning, with a “stormy” phase, while during childhood seizures are usually well-controlled with ASMs. Response to ASMs may be associated with a slight cognitive improvement in some cases, although most patients still suffer from moderate to profound ID throughout their lives. In more severe cases, feeding difficulties, failure to thrive with increased potentially fatal comorbidities may be observed.

The role of heterozygous variants remains to be fully elucidated. Future studies should address the functional impact of heterozygous *CNTNAP2* variants and the related pathomechanisms with ultimately important implications for patient management and counselling.

## Supplementary Information

Below is the link to the electronic supplementary material.

Supplementary file1 (XLSX 57 KB)

## Data Availability

The datasets generated during and/or analyzed during the current study are available from the corresponding author. *CNTNAP2* variants have been submitted to ClinVar.
